# Circulating miRNAs as Potential Biomarkers Distinguishing Relapsing–Remitting from Secondary Progressive Multiple Sclerosis. A Review

**DOI:** 10.3390/ijms222111887

**Published:** 2021-11-02

**Authors:** Sylwia Pietrasik, Angela Dziedzic, Elzbieta Miller, Michal Starosta, Joanna Saluk-Bijak

**Affiliations:** 1 Department of General Biochemistry, Faculty of Biology and Environmental Protection, University of Lodz, Pomorska 141/143, 90-236 Lodz, Poland; sylwia.pietrasik@edu.uni.lodz.pl (S.P.); joanna.saluk@biol.uni.lodz.pl (J.S.-B.); 2 Department of Neurological Rehabilitation, Medical University of Lodz, Milionowa 14, 93-113 Lodz, Poland; elzbieta.dorota.miller@umed.lodz.pl (E.M.); michal.starosta@umed.lodz.pl (M.S.)

**Keywords:** circulating microRNA, relapsing–remitting multiple sclerosis, secondary progressive multiple sclerosis, biomarker, neuroinflammation

## Abstract

Multiple sclerosis (MS) is a debilitating neurodegenerative, highly heterogeneous disease with a variable course. The most common MS subtype is relapsing–remitting (RR), having interchanging periods of worsening and relative stabilization. After a decade, in most RR patients, it alters into the secondary progressive (SP) phase, the most debilitating one with no clear remissions, leading to progressive disability deterioration. Among the greatest challenges for clinicians is understanding disease progression molecular mechanisms, since RR is mainly characterized by inflammatory processes, while in SP, the neurodegeneration prevails. This is especially important because distinguishing RR from the SP subtype early will enable faster implementation of appropriate treatment. Currently, the MS course is not well-correlated with the biomarkers routinely used in clinical practice. Despite many studies, there are still no reliable indicators correlating with the disease stage and its activity degree. Circulating microRNAs (miRNAs) may be considered valuable molecules for the MS diagnosis and, presumably, helpful in predicting disease subtype. MiRNA expression dysregulation is commonly observed in the MS course. Moreover, knowledge of diverse miRNA panel expression between RRMS and SPMS may allow for deterring disability progression through successful treatment. Therefore, in this review, we address the current state of research on differences in miRNA panel expression between the phases.

## 1. Introduction

Multiple sclerosis (MS) is an autoimmune disease characterized by chronic inflammation, demyelination, and neurodegeneration of the central nervous system (CNS) [1]. According to the latest data, the estimated number of people who suffer from MS in 2020 was 2.8 million worldwide (35.9 per 100,000 population) [2]. Just as in other autoimmune diseases, MS is found to be more prevalent in women than in men (female:men ratio 3:1) [3]. The disease is most often diagnosed in young adults between 20 and 40 years of age, who are in the period of the greatest activity in life; however, there are cases of the disease onset both in children (2–10% of patients being younger than 18 years) and older people (near 4% after age 50) [4,5]. MS is generally most prevalent in northern geographic latitudes, and it is suggested to be mainly due to the influence of environmental, genetic, and ethnicity, as well as behavioral differences [6,7].

The first neurological incident suggesting MS is known as a clinically isolated syndrome (CIS), which lasts at least 24 h and is characterized by symptoms and signs indicating either the presence of a single lesion (monofocal episode) or several lesions (multifocal episode) within the CNS. Patients who experience CIS may or may not develop MS [8]. Most people who suffer from MS (85–90%) are diagnosed with relapsing-remitting multiple sclerosis (RRMS), portrayed by active bouts of disease followed by a relative stabilization until the next relapse. Eventually, after 10–15 years from disease onset, more than 70% of RRMS patients transit to secondary progressive multiple sclerosis (SPMS), characterized by steadily increasing neurological disability, independent of relapses. The remaining 10–15% of MS patients develop primary progressive multiple sclerosis (PPMS), characterized by slowly worsening symptoms from the beginning, without prior or intermittent exacerbations and remissions [9].

This is the result of its very heterogeneous, multifactorial etiopathogenesis and unpredictable disease course, in which several clinical phenotypes with distinct underlying pathogenic mechanisms can be distinguished [9,10,11,12]. Another difficulty in the form of a varied response to applied therapies among patients additionally aggravated the challenges doctors face [13].

The pathophysiology of MS is a complex autoimmunological process characterized by the progressive loss of neurological function caused by the destruction of the axonal myelin sheath in several areas of the brain and the spinal cord [14]. MS is believed to be driven by systemic immune activation of autoimmunity against CNS components. In MS, the inflammatory state is mediated by the interaction between several immune cells, such as T and B cells, macrophages, CNS glial cells (microglia and astrocytes), as well as antigens reactive against myelin, including myelin basic protein (MBP) and myelin oligodendrocyte glycoprotein (MOG) [15]. The commonly implicated myelin-reactive CD4+ cells in the pathophysiology of MS are Th1 and Th17 lineage, defined on the basis of the production of interferon-γ (IFN-γ) and interleukin-17 (IL-17), respectively [16]. Autoreactive Th cells and activated monocytes secrete elevated amounts of proteolytic enzymes, such as matrix metalloproteinases (MMPs), which can degrade tight junction proteins and cause blood–brain barrier (BBB) disruption [17]. Moreover, Th1 and Th17 cells can cross the BBB and migrate to the CNS, followed by microglia activation and secretion of pro-inflammatory cytokines, such as tumor necrosis factor α (TNF-α), IL-1β, and IL-6 [18].

The interplay between relapses and progression during MS has led to the split of the disease into two leading phases, which are characterized by different but still mutually interacting pathological processes within the CNS [19]. In the early RRMS, the critical mechanism of the initiation of the disease process is inflammation and BBB damage. Patients with the RR subtype display stronger inflammatory features than progressive forms (PP and SP subtypes) [20]. In the advanced stages of the disease, the ongoing inflammation process gradually contributes to neurodegeneration, which seems to dominate in progressive MS [21]. SPMS is manifested by predominant neurodegeneration, brain atrophy, and steady clinical exacerbation, even when a patient is not experiencing a relapse [22]. Since therapies mainly exert a dampening effect on the immune system, this may be one explanation as to why therapeutic effects are poor in progressive MS. Moreover, many disease-modifying therapies (DMTs) used in RRMS are ineffective or even harmful for SPMS patients, thus highlighting the need to modify the therapeutic interventions used [23,24,25,26].

Currently, diagnosis of the conversion from RRMS to SPMS is based only on retrospective clinical and radiological assessment. However, the retrospective assessment presents many difficulties in terms of establishing the time point of disease progression [27]. Lublin et al. proposed a one-year retrospective clinical assessment of disability progression, as measured by the expanded disability status scale (EDSS), as diagnostic criteria for SPMS [28]. Nevertheless, disability progression in SPMS is not frequently clearly measurable by clinical scales. Moreover, EDSS is widely criticized for its lack of linearity, over-reliance on inferior limb function and ambulation, low sensitivity, and high interrater variability [29].

According to the 2017 McDonald’s criteria, the primary tests for MS diagnosis are magnetic resonance imaging (MRI) findings and cerebrospinal fluid (CSF) examination. Indeed, oligoclonal band (OCB) analysis, despite being an invasive diagnostic method linked to the lumbar puncture, is a recommended analysis in CSF samples, while basic CSF biochemistry, as well as tests for intrathecal immunoglobulin G (IgG) index, are not recommended [30]. At the early MS stage, the volume of demyelinating lesions is superior in the white compared to the gray matter, whereas progressive MS manifests by widespread gray matter demyelination. It was shown that focal gray matter atrophy is an untimely indicator of progression, and the pace of gray matter atrophy correlated with MS development [9,31,32,33]. Nevertheless, standard MRI-based imaging does not fully reflect the ongoing disease mechanisms, such as demyelination/remyelination, microglial activation, astrogliosis, as well as neurodegeneration, which contribute to subclinical disease activity [34].

The available literature data report the existence of biologically active molecules that could be a potentially helpful tool for differentiating the RR phase from SP. Pasquali et al. reported that the plasmatic levels of proinflammatory cytokines, both IFN-γ and IL-17, are higher in RRMS compared to SPMS patients, while the level of transforming growth factor-β (TGF-β), a molecule with immunosuppressant activity, was much lower in RRMS in comparison to SPMS [35]. Another group of active molecules indicated in the literature are light neurofilaments (NFL) and glial fibrillary acid protein (GFAP), a marker of astrocyte damage and astrogliosis, in serum [36,37]. Högel et al. claimed that GFAP and NFL levels in serum are higher in patients with SPMS than RRMS, as well as correlate with a higher EDSS parameter [38]. Whereas Ayrignac et al. demonstrated higher levels of both NFL and GFAP in serum from PPMS compared to RRMS, indicating that they might be markers of the disease progression [37]. Based on the above studies, we consider NFL and GFAP as potential progressive MS and RRMS distinguishing biomarkers.

Most importantly, the majority of autoimmune diseases are accompanied by inflammation, which is why it is strongly recommended not to take inflammatory factors into account as proper MS markers.

Therefore, it is imperative to identify suitable diagnostic tools, for instance, in the form of sensitive, reliable, and stable biomarkers that can help distinguish the clinical phenotypes of MS, predict disease progression, and provide a correlation with disability [33]. It is firmly not recommended to consider cytokines/chemokines measured in serum/plasma as a reliable marker, especially because they are highly non-specific to concrete disease entity [39]. Therefore, the inflammatory markers mentioned above can only complement MRI and patients’ clinical characteristics [9]. Recent studies have demonstrated that altered expression of some miRNAs may serve as valuable biomarkers to diagnose MS, and rapidly and effectively distinguish RR from the SP phase [40,41].

## 2. Biogenesis and Characteristics of miRNA

In the past few years, numerous studies have confirmed the essential role of small (19–25 nucleotides) non-coding RNA molecules, called microRNAs (miRNAs), as significant regulators of biological processes associated with the pathophysiology of various autoimmune and neurodegenerative disorders, including MS [42,43,44,45]. MiRNAs are remarkably stable, resistant to endogenous RNase activity, simple to obtain, and above all, highly sensitive to the processes taking place in the organism [46]. Furthermore, microRNAs have distinct expression level patterns, which could be characteristic of the specific disease [47]. Those features have made circulating miRNAs a potentially promising prognostic biomarker, being investigated for various human disorders, including neurodegenerative diseases and other neurological pathologies [48]. Despite those analyses, no diagnostic miRNA has been effectively applied in clinical examination until now. Still, more research regarding miRNA activity in MS, especially at the genetic level, needs further clarification. The analysis of the expression level of miRNAs potentially involved in neurodegeneration processes might provide new knowledge of MS etiopathogenesis and might be helpful while choosing the proper therapy used in different stages of the disease.

The formation of miRNA consists of several steps [49]. Gene-encoding miRNA is transcribed by RNA polymerase II, which leads to the formation of a long primary transcript, termed primary miRNA (pri-miRNA). In the nucleus, the pri-miRNA is cleaved by a microprocessor complex of the RNase III endonuclease Drosha with DiGeorge syndrome critical region 8 (DCGR8) protein into pre-miRNA. Next, pre-miRNA is transported to the cytoplasm by exportin-5, where it is cleaved by the RNase III endonuclease Dicer to produce an RNA duplex [50]. Finally, the mature miRNA is transferred to Argonaute family proteins (Ago) in the RNA-induced silencing complex (RISC) core. One strand of the duplex (-3p) is typically degraded by Ago. The other strand (-5p) is loaded into RISC (usually, this strand is the one with lower thermodynamical stability at the 5′end) [51]. However, both strands (-5p and -3p) may be loaded into RISC at similar frequencies for some miRNAs. In fact, while RISC loading may strongly favor the incorporation of one strand (-5p), recent research has demonstrated a small fraction of the -3p strand loaded into RISC for essentially all miRNA families [52].

Together with the RISC, single-stranded miRNA, using as few as 6–8 nucleotides (so-called ‘seed region’) which are located at the 5′end, hybridizes to the complementary sequences of the target messenger RNA (mRNA) in the 3′UTR (untranslated region), known as a miRNA response element (MREs). The hybridization results in specific post-transcriptional gene silencing—RNA interference (RNAi) [53]. Moreover, the interaction of miRNAs with other regions, including coding sequence and 5′UTR, has also been reported [54].

The effects of RNAi depend on the grade of the complementarity of miRNA with the target mRNA. Excellent complementarity leads to the destabilization and degradation of the mRNA transcript by the catalytic Ago2 protein. Whereas, if the complementarity is not full (this effect is more often), it results in translational repression [55]. In this way, miRNAs modulate more than 60% of all mammalian mRNAs, and therefore a wide range of cellular processes, including development, differentiation, proliferation, metabolism, and apoptosis [56]. Furthermore, there is a complex network of mutual interactions between miRNAs and mRNAs—one miRNA can bind to hundreds of target mRNAs, and a single mRNA can be targeted by various miRNAs [57]. This means that the combination of multiple miRNAs determines the expression of the same gene, and the lack of a single regulator can be compensated by other miRNA molecules complementary to the same target transcripts [58,59].

MiRNA expression profiling is conducted using a set of various methods, such as miRNA microarray platforms, quantitative real-time polymerase chain reaction (qRT-PCR), digital PCR (dPCR), in situ hybridization, and high-throughput sequencing (next-generation sequencing, NGS). Nowadays, the NGS technique is most frequently used to profile miRNA in distinct sets of diseases. It is noteworthy that each of these methods provides a large amount of information about miRNAs profile in different diseases; nevertheless, every method also has some disadvantages. Although NGS is challenging due to cost, labor, time consumption, and professional bioinformatics support for data analysis, it profiles both known and unknown miRNAs, which are beyond the capabilities of qRT-PCR and microarrays. Microarrays require a higher concentration of miRNAs and have lower specificity than qRT-PCR. On the other hand, sensitive and specific qRT-PCR, the standard technique to measure miRNA expression, provides medium-throughput concerning the number of samples processed per day [60,61,62,63]. Furthermore, quantification of samples with low concentrations of nucleic acids using this method can be challenging, while dPCR technology enables absolute quantification through partitioning the reaction. dPCR, highly sensitive and accurate in molecular detection, has demonstrated applications such as trace DNA detection, rare mutation detection, and copy number variation. Unfortunately, its disadvantages compared to qPCR are narrow dynamic range and high cost [64].

It was demonstrated that circulating miRNAs present in various bio-fluids, such as saliva, blood, plasma, serum, and CSF, can be released or produced due to different events, including (1) passive leakage from damaged cells due to chronic inflammation, apoptosis, or necrosis, (2) active secretion through cell-derived microparticles, exosomes, shedding vesicles, and apoptotic bodies, and (3) active transport by a complex with protein, such as Ago2 [65]. Furthermore, exosomes can provide an excellent source of miRNA biomarkers because of their easy extraction from body fluids [66]. Circulating miRNAs enclosed in membrane vesicles are extremely stable in the extracellular environment, with high RNAse activity [67]. High stability means that circulating miRNAs are long-lived in bio-fluids and are consequently proposed as attractive diagnostic [68] and prognostic biomarkers [69,70]. Some experimental studies also demonstrated strong resistance of endogenous circulating miRNAs for stressful conditions, such as repeated freeze–thaw cycles, temperature differences, and prolonged storage time, which are favorable traits for their analyses [71,72]. miRNAs circulating in the human body have the potential for being used as multi-marker models that could be non-invasive, less expensive, and less time-consuming methods in monitoring the disease course and its response to treatment than the classical protein markers used so far [73]. This is due to the simplicity of their obtaining, high sensitivity, and specificity for a given disease entity [74].

According to the miRNA repository, miRbase (www.mirbase.org; accessed date: 21 September 2021) currently lists 1917 precursors and 2654 mature miRNAs in *Homo sapiens*, which control the expression of hundreds of different genes [75]. The understanding of which miRNAs or panels are dysregulated in particular diseases makes miRNAs emerge as valuable therapeutic targets [76]. The human miRNA-associated disease database (HMDD) (www.cuilab.cn/hmdd; accessed date: 21 September 2021) operates as a rich resource for scientists, screening the permanently growing number of miRNA profiles for a broad range of diseases.

## 3. MiRNAs as Potential Biomarkers in MS

At the time of writing this review, the HMDD covered 148 different miRNAs associated with MS pathogenesis, predisposing to a disease marker [77].

**Figure 1:**
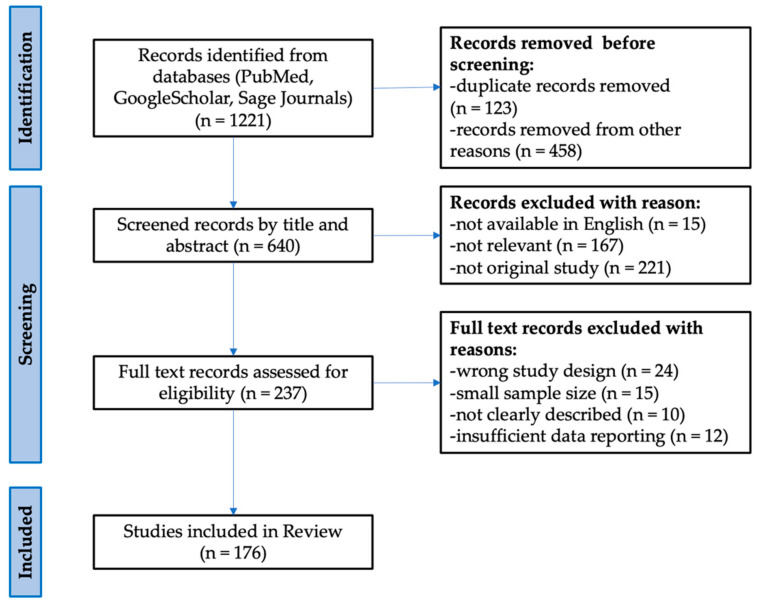
Flow diagram representing the study selection process based on the PRISMA template.

Using a literature search for peer-reviewed articles, conducted through the PubMed and Sage Journals databases, we have retrieved and compared all significantly different circulating miRNAs between RRMS and SPMS. Google Scholar was also used to seek open-access articles. The data from the World Health Organization (WHO) and clinicaltrials.gov websites were also considered in the above review. The review consists of 176 literature positions, including 98 original research (case reports, clinical trials, cohort studies), 73 reviews (systemic reviews, literature reviews, and meta-analyses), 1 website, and 4 chapters in books. The cited works are in the 1982 to 2021 range of years. Identified relevant reviews were hand-searched for additional relevant references. The following search terms were used to locate articles specific to this study: relapsing–remitting multiple sclerosis, secondary progressive multiple sclerosis, biomarkers, circulating miRNAs, plasma, serum, differences, and expression. Variations of these terms were used to ensure exhaustive search results. After identifying all the keywords, synonyms, and phrases, the Boolean operators “AND” and “OR” were used. The PubMed search was performed using terms and database-appropriate syntax: “expression” AND “differences” AND “plasma” OR “serum” AND “circulating miRNAs” OR “miR-92a-1-3p” OR “miR-633-5p” OR “miR-485-3p” OR “miR-337-3p” OR “miR-326-5p” OR “miR-30b-5p” OR “miR-27a-3p” OR “miR-223-3p” OR “miR-181c-5p” OR “miR-145-5p” OR ”let-7c” OR “let-7d” AND “biomarkers” AND “relapsing–remitting multiple sclerosis” OR “secondary progressive multiple sclerosis”. Furthermore, the search terms relating to “multiple sclerosis” were not useful as the search facilities were not precise. Based on the PRISMA (Preferred Reporting Items for Systematic Reviews and Meta-Analyses) template, a diagram representing the literature strategy was created (Figure 1) [78].

It is evident that miRNAs are recognized as essential modulators of inflammatory responses; however, against intuition, the activity of miRNAs is not only restricted to a simple inhibition of inflammation. The earlier discoveries of a handful of miRNAs involved in inflammatory responses have encouraged researchers to focus their studies on their systematic profiling in different cells and cell-free biological fluids to understand their function in regulating the inflammatory process [79]. Recent studies have uncovered a significant role for microRNAs as regulators of major cellular functions, such as development, differentiation, growth, and metabolism. Due to their abilities, microRNAs are believed to be implicated in many human pathologies, including inflammatory, autoimmune, and neurodegeneration, all characteristics of MS [80,81,82,83]. What is more, miRNAs modify the transcripts for proteins engaged in remyelination, neurogenesis, and gliogenesis [84,85,86,87].

In the literature are emerging several circulating miRNAs being either up- or down-regulated in MS patients vs. healthy controls (HCs) and differently expressed in various disease subtypes [88]. Dysregulated miRNAs in MS obtained from different tissues and cell types were analyzed, for instance, in a comprehensive review by Piket et al. or Gandhi [89,90]. Piket et al. provided a detailed overview of 61 studies that examined miRNAs in MS (most of them analyzed RRMS vs. HCs). They focused on the mechanisms of the most dysregulated miRNAs and also used predicted targets of the most dysregulated miRNAs to highlight affected pathways. From their research, it resulted that the prime affected pathway was TGF-β signaling, which is important in the differentiation and function of Th17 and Treg cells. Moreover, some of the miRNAs which can be found in their comprehensive overview overlap with miRNAs which we found dysregulated in RRMS vs. SPMS, including let-7d, 181c, or 145-5p [89]. Otaegui et al. indicated that cell-derived miRNAs are promising biomarkers in MS and selected those that may be indicators of the relapse and remission phases [91]. It was also shown that circulating miRNAs help distinguish MS subtypes [92]. Furthermore, other miRNA analyses have been performed between MS subtypes (Vistbakka et al. refers to PPMS vs. SPMS, Mandolesi et al. refers to CIS/Radiologically Isolated Syndrome vs. RRMS vs. progressive MS, or Perdaens et al. refers to relapsing and remitting MS vs. HCs) [88,93,94]. The panel of miRNAs with disturbed expression in the course of MS obtained from different bio-fluids, such as CSF, whole blood, plasma, as well as T- and B-cells, which includes, e.g., let-7, miR-320, miR-27a, miR-223, miR-155, and their target genes, has been found [95]. One of the more thoroughly studied miRNAs in the context of MS pathogenesis is miRNA-155, for which the pro-inflammatory effect is associated with activation of inflammatory cells, increasing permeability of the BBB, phagocytosis of myelin by macrophages, and neurodegenerative processes [96,97]. Circulating miR-155 contributes to microglial activation and polarization of astrocytes towards the neurotoxic A1 phenotype, thereby driving demyelination processes [98,99]. Additionally, miR-155, by having a suppressive effect on mothers against decapentaplegic homolog 2 (SMAD2) and SMAD4, leads to upregulation of the Nogo receptor (NgR), which is engaged in the suppression of neuronal growth. Hence, upregulated miR-155 may induce demyelination [100,101]. MiR-155 also has a huge impact on the development of neuropathic pain and indirectly influences a Treg cell differentiation involved in alleviating pain hypersensitivity [102]. Another documented miRNA engaged in neuroinflammatory processes, which could have an effect on MS, is let-7a. As proven by Kacperska et al., this miRNA revealed a statistically significant difference in the expression in patients’ plasma (downregulated in RRMS vs. HCs (*p* = 0.002)), whereas Gandhi et al. showed a statistically significant difference in the expression of let-7a in the plasma obtained from SPMS and the HCs (*p*  =  0.002); however, no significant differences were reported for the RRMS vs. HC comparison [103,104]. Let-7a serves as an essential modulator of neuroinflammatory processes, inhibits apoptosis, and promotes the neuroprotective M2 phenotype in microglia under active inflammatory conditions, as a result being involved in the anti-inflammatory process [105]. In response to inflammation, upregulated circulating let-7a represses the production of IL-6 and promotes the expression of brain-derived neurotrophic factor (BDNF) and anti-inflammatory cytokines, such as IL-4 and IL-10 in microglia [106]. IL-4′s role in regulating inflammation within the CNS was demonstrated in the experimental autoimmune encephalomyelitis (EAE), an animal model of MS, in which mice deficient in IL-4 exhibited more severe EAE clinical disease [107]. Another huge miRNA family involved in MS pathology is the miR-320 family (miR-320a, miR-320b, miR-320c, miR-320d, and miR-320e). Studies on the mice with EAE showed that overexpression of cell-free miR-320 isoforms plays a possible role in the autoimmune neuroinflammation and the pathogenesis of MS disease. Talebi et al. found that miR-320 isoforms could target SMAD2 and TGFBR2 (TGF-β Receptor 2), thereby diminishing the TGF-β signaling pathway [108]. This pathway contributes to the increasing differentiation of naive T cells into Treg cells and inhibition of Th1, leading to the decrease in IFN-γ production and lessening EAE [109]. As a result, overexpression of miR-320 isoforms might be involved in the neuroinflammation and pathogenesis of MS through reducing neuroprotective and immunomodulatory effects of TGF-β [108]. Moreover, in B cells of MS patients during a disease relapse, the expression of miR-320a is decreased, leading to increased MMP9 expression, being a marker of disease activity in patients with MS [110,111]. An elevated level of MMP9 can be implicated in the pathogenesis of MS, as this enzyme is involved in BBB degradation, thereby intensifying neuroinflammation and worsening the disease course [112].

In 2016, the clinical trial, which was based on a pilot study that seeks to characterize differences in miRNA profiles and cell products obtained from blood and CSF of patients in the early (CIS) and later (SP) stage of MS, as well as healthy participants (with no neurological or autoimmune illness), was completed. Although no results have been published, the aforementioned study is particularly promising, taking into account that obtained miRNA panels could then be correlated to clinical manifestations and subtypes of MS [113].

## 4. Potential Candidates for a Panel Distinguishing the RR from SP Phase

The use of miRNAs as biomarkers in MS is still being developed. Until now, in the case of differences between RRMS and SPMS, only a few reported studies have analyzed circulating miRNA in cell-free biological fluids.

Recently, it has been a trend to use a panel of multiple miRNAs, more favorable than a single miRNA, focusing on early diagnostic and/or prognostic biomarkers in many diseases [114,115,116]. Using a specific panel of several miRNAs increases the credibility of the obtained results and reduces the risk of a false-positive diagnosis to a minimum.

The study conducted by Haghikia et al. showed that miR-181c (*p* = 0.02) and miR-633 (*p* = 0.0005) are downregulated in the CSF of SPMS when compared to RRMS [117]. Kramer et al. confirmed that those miRNAs are dysregulated in the CSF in MS. However, their study claimed that miR-181c is upregulated in SPMS compared to RRMS (*p* = 0.036) and miR-633-5p does not reach the statistically significant differences (*p* = 0.468) [118]. It was reported that this miRNA could predict the conversion from CIS to RRMS, and the enhanced level of miRNA-181c in the CSF could be the early marker of the highly active phase of MS [119]. There is an experimental study on rat cortical neurons, which demonstrated the involvement of miR-181c-5p in the regulation of neuronal maturation and synaptogenesis in the cortex and the molecular responses of astrocytes under inflammatory conditions [120]. What is more, several target genes were identified for miR-181c-5p, including SMAD7 (a negative regulator of TGF-β signaling) engaged in Th17 differentiation, being a major driver of CNS autoimmunity in MS [121]. Nevertheless, no targets for miR-633-5p have been validated so far. However, the bioinformatics prediction tool, TargetScan, revealed a few potential binding sites, including macrophage scavenger receptor 1 (MSR1), which is also discussed in the context of TGF-β-induced microglia toxicity [118].

The study of Gandhi et al. explored the potential of miRNA profiling to distinguish subtypes of MS. Their research based on qRT-PCR analysis showed that expression of miR-92a-1-3p, let-7c, let-7d, and miR-145-5p is significantly upregulated in plasma from RRMS in comparison to SPMS (*p* = 0.022, *p* = 0.04, *p* = 0.047, and *p* = 0.01, respectively) [104]. In the multivariate model, miR-92a-1-3p showed a significant association with RRMS (adjusted OR = 1.35, *p* = 0.022), whereas it demonstrated no link with SPMS. Furthermore, they showed a positive correlation between miR-92a-1-3p expression and EDSS and disease duration, which may indicate that miR-92a-1-3p may be an early marker of disease progression [104]. In the same study, the additional biostatistical analysis showed that miR-92a-1-3p targets genes which regulate the CD40-CD40L pathway [104]. CD40-CD40L dyad was found to be essential in modulating the aberrant inflammatory response in MS. Although CD4+ and CD8+ T cells are extensively present during immune activation and can express CD40L, in MS, CD40L expression is only detected on CD4+ T cells, not on CD8+ T cells [122]. CD40L is neither identified in the CNS of healthy people nor patients with other neurodegenerative diseases [123]. CD40–CD40L interactions can promote the inflammatory response underlying MS, whereas it is confirmed that inhibition of the CD40-CD40L pathway reduces disease severity in the EAE [124]. Additionally, Balashov et al. demonstrated that CD40L-dependent Th1 differentiation and immune activation were only observed in the progressive MS but not in the RRMS, suggesting a link to disease pathogenesis and progression and providing a basis for immune response intervention in the disease [125]. The data considered above suggest that upregulated miR-92a-1-3p expression in RRMS via the CD40-CD40L pathway may be a genuine marker of early inflammation burst induced by CD4+ T cells, both in MS and EAE. MiR-92a-1-3p is involved in the pathogenesis of EAE, and it might function as a positive regulator of T cell differentiation towards pathogenic Th1 cells [126].

Subsequent miRNAs from the let-7 family, apart from let-7a, differently expressed in MS, are let-7c and let-7d. Banerjee et al. suggested an important role of let-7c in regulating macrophage polarization derived from bone marrow cells of mice. They found that overexpression of let-7c in macrophages promotes their polarization into the anti-inflammatory M2 phenotype, possibly by targeting the C/EBP-δ transcription factor, which plays a vital role in the inflammatory response [127]. Kim et al. conducted experiments on mice with a dendritic cell (DC)-specific deletion of the transcriptional repressor B lymphocyte-induced maturation protein1 (Blimp1), which showed that the increased level of let-7c that is present when Blimp1 expression is low results in a proinflammatory DC phenotype [128]. Besides, toll-like receptor 7 (TLR7), a noncatalytic receptor protein present in macrophages and DCs involved in innate immunity and inflammation, recognizes let-7c and plays a negative role in controlling neuronal growth in cultured neurons. Thereby, let-7c might suppress the dendritic growth of cortical neurons [129]. It is worth emphasizing that neuronal innate immune responses may influence neurodevelopment and neurodegeneration through the regulation of neuronal morphology [129]. Let-7d is predicted to target IL-10 since there is a strong positive correlation between the pro-inflammatory cytokine IL-1B and let-7d [130]. This is important, taking into consideration that IL-10 has significant regulatory effects on immunological and inflammatory responses, for instance, because of its capacity to inhibit the production of proinflammatory cytokines by monocytes [131].

In the next study, Regev et al. found that the miR-27a-3p was the only miRNA out of 652 analyzed miRNAs that differentiated progressive patients from relapsing ones. Expression of miR-27a-3p in serum was significantly upregulated in the RRMS compared to SPMS. AUC for miR-27a-3p was high and reached a value of 0.78 [92]. It was also demonstrated that miR-27a-3p expression shows a strong link to the neurotrophin signaling pathway [92]. In CNS, neurotrophins, which play a protective role for neuronal circuitry, facilitate synaptic transmission, and regulate brain plasticity, are essential for memory and regenerative processes [132]. It was shown that reduced production of neurotrophic factors, such as BDNF in patients with SPMS, can contribute to the progression of demyelinating disease and axonal loss [133]. Probably, miR-27a-3p, through regulation of the neurotrophin pathway, may be an early marker of neurodegeneration in patients with late RRMS and play a role as the prognostic marker of RR-to-SP transition. MiR-27a-3p targets many proteins of intracellular signaling networks, which regulate, for example, the activity of nuclear factor kappa-light-chain-enhancer of activated B cells (NF-κB) and mitogen-activated protein kinases (MAPKs), which control numerous cellular events associated with the inflammatory response, apoptosis, and cell proliferation [134,135]. Lu et al. revealed that downregulation of miR-27a-3p inhibits the inflammatory response (reduced the expression levels of pro-inflammatory cytokines, such as IL-1β, IL-6, and TNF-α) and hippocampal neuronal cell apoptosis by targeting mitogen-activated protein kinase 4 (MAP2K4) in epilepsy. Thus, increased expression of miR-27a-3p may induce inflammatory burst and hippocampal neuronal apoptosis by targeting MAP2K4 [136]. Ahmadian-Elmi et al. suggested that high expression of miR-27a-3p can suppress the TGF-β signaling pathway by directly targeting some key transcription factors, such as SMADs and Cul1 proteins [137]. It was shown that the SMAD2/3 complex and SMAD4 can regulate forkhead/winged-helix transcription factor 3 (FOXP3) gene expression [138]. FOXP3 is especially involved in the development and function of Treg cells, and it can inhibit the expression of the IL-17A gene by interacting with retinoic acid receptor-related orphan receptor-γt (RORγt), a transcription factor that directs the differentiation of inflammatory Th17 cells [139]. In RRMS patients, silencing SMADs and Cul1 by upregulated miR-27a-3p may block the TGF-β signaling pathway, leading to the suppression of naïve T cell differentiation into Treg cells. Finally, reducing the Treg number and enhancing the number of Th17 cells in the relapsing phase will promote inflammation in the RR stage of MS [137]. Additionally, miR-27a-3p is a regulator of oligodendrocytes’ development and survival. Increased miR-27a-3p level leads to inhibition of oligodendrocytes differentiation/maturation in mice and human oligodendrocyte progenitor cells and myelination and remyelination in vivo in mice. Therefore, decreasing the levels of miR-27a-3p following demyelination is critical for facilitating remyelination [140].

In another study, Regev et al. reported that miR-337-3p in serum was significantly downregulated in SPMS compared to RRMS in one of the cohorts (*p* = 0.01), while no significant differences were found for the other cohorts [141]. Moreover, its increased expression negatively correlated with the EDSS in three independent MS cohort studies. Thus, it may be accepted that miR-337-3p might be a potential biomarker candidate for disability and disease progression [141]. Of interest, it was demonstrated that miR-337-3p targets Ras-related protein 1 (Rap1) A protein, which is a well-established major component of the integrin activation pathway, hence indicating a potential role of miR-337-3p to serve as a biomarker for predicting the therapy response to natalizumab (an α4β1-integrin inhibitor) in MS patients [142]. Additionally, Rap1 signaling impacts upon autoimmune T cells at various levels and confirms the concept that sustained Rap1 activation diminishes T cell-mediated autoimmunity. Therefore, miR-337-3p via Rap1 signaling may initiate the pathogenic character of T cells in immune-mediated inflammatory diseases, such as MS [143].

There is also Sharaf-Eldin et al.’s study, which is promising, however, it requires future verification on a larger number of patients and detailed validation. Sharaf-Eldin et al. carried out a study on miR-145-5p, miR-223-3p, and miR-326-5p, and concluded that only miR-326-5p indicated a statistically significant difference (*p* = 0.018) between RRMS and SPMS patients (overexpression in RRMS vs. SPMS). Additionally, combinations of miR-145-5p + miR-326-5p, miR-223-3p + miR-326-5p, and miR-145-5p + miR-223-3p + miR-326-5p can differentiate RRMS from SPMS, with the area under the curve (AUC) and 95% confidence intervals (95% CI) values of (0.737 (0.57–0.904), *p* = 0.014), (0.713 (0.531–0.896), *p* = 0.027), and (0.772 (0.619–0.925), *p* = 0.005), respectively [144]. AUC is a parameter providing an estimate of the miRNA’s ability to discriminate the groups compared, known as an area under the receiver operating characteristic curve [145]. Kornfeld et al. demonstrated that miR-145-5p targets myelin gene regulatory factor (MYRF), a transcriptional regulator required for CNS myelination and oligodendrocyte maturation. This was confirmed by the fact that mice lacking MYRF display severe neurological abnormalities and severe deficits in myelin gene expression [146]. Studies on transient middle cerebral artery occlusion in rats indicated that miR-145 plays a role in the brain’s antioxidant defense because its lower expression led to increased protein expression of superoxide dismutase-2 (SOD2), one of the major antioxidants [147]. Moreover, miR-145-5p was identified as a putative regulator of nuclear receptor subfamily 4 group A member 2 (NR4A2), also known as Nurr1 [148]. The research performed on the secondary spinal cord injury in the rat model indicated that miR-145-5p inhibition decreases inflammation and oxidative stress, which, together with mitochondria dysfunction, feature prominently in MS [149], by targeting Nurr1 to block TNF-α signaling [150].

It was reported that miR-223-3p is involved in regulating hematopoiesis, myeloid progenitor proliferation, granulocyte differentiation, and thereby immune response [151]. Studies on the EAE model suggested that miR-223-3p has an important role in the development of CNS inflammation. MiR-223-3p regulates myeloid DC-induced activation of pathologic Th17 responses during autoimmune inflammation, controlling IL-1β, IL-6, and IL-23 cytokines [152]. Mice with miR-223–3p-knockout exhibited reduced numbers of myeloid DCs and Th17 cells in the CNS, thereby reducing EAE disease severity linked to decreased inflammation [152]. Besides, miR-223 is required for efficient macrophage M2 polarization, and mice lacking miR-223 display impaired CNS remyelination [153]. Depending on the type of regulated pathway, miR-233-3p exhibits anti- and pro-inflammatory properties. On the one hand, Li et al. demonstrated that miR-223-3p modulates the noncanonical NF-κB (nuclear factor-κB) pathway by targeting transcripts of the inhibitor of kappa B kinase alpha (IKKα) (engaged in activation of NF-κB), which is an anti-inflammatory factor that may prevent the spontaneous activation of macrophages, thus promoting controlled inflammation in the human myeloid leukemia cell line [154]. On the other hand, miR-223-3p, by targeting TNF receptor-associated factor 6 (TRAF6) and TGF-β activated kinase 1 binding protein 1, suppresses the canonical NF-κB pathway. Therefore, miR-223-3p expression was indicated to decrease neutrophil activation, suggesting anti-inflammatory effects [155]. Furthermore, Chen et al. demonstrated that miR-223-3p directly targets the transcription factor signal transducer and activator of transcription 3 (STAT3). It was shown that miR-223-3p overexpression was associated with a significant decrease in STAT3 levels and reduction in the production of IL-1β and IL-6, but not TNF-α, in macrophages. Thereby, miR-223-3p might regulate processes associated with the regulation of inflammatory responses in macrophages [156]. In addition, miR-223-3p could downregulate the nod-like receptor pyrin domain containing 3 (NLRP3), which is thought to be a critical and necessary factor in MS development. Decreasing the NLRP3 level inhibits inflammation through caspase-1 and IL-1β, thus reducing brain edema and improving neurological functions [157,158,159]. Besides, miR-223-3p appears to promote neuronal protection partly through the regulation of glutamate receptor signaling. Glutamate receptor 2 and N-methyl D-aspartate receptor 2B expressions are subunits for glutamate’s ionotropic transmembrane receptors that mediate fast synaptic transmission in the CNS. It was demonstrated that miR-223-3p overexpression in the retina and optic nerve, by reducing the expression of the above subunits, had blocked the formation of EAE-driven pathological axonal swellings, which are attributed to excitotoxicity of glutamate [160,161].

It was investigated that miR-326-5p downregulates the expression of transcription factor Ets-1 [162], a negative regulator of Th17 differentiation, thereby promoting Th17 differentiation. MiR-326-5p expression is closely correlated with disease severity, both in patients with MS and mice with EAE [162]. Nevertheless, the exact role of Ets-1 in regulating the differentiation and function of Th17 cells still remains unknown [163]. In another study, Honardoost et al. also confirmed the potential of miR-326-5p, obtained from peripheral blood lymphocytes, as a diagnostic biomarker to discriminate between relapsing and remitting phases of MS disease [164]. Studies performed on phosphatase and tensin homolog-induced kinase 1 (PINK1)-deficient mice suggest that miR-326 may upregulate GFAP expression during neural stem cells’ (NSCs) differentiation and brain development [165]. In addition, Junker et al. showed that miR-326, upregulated in active MS lesions, targets the 3′-UTR of CD47 in brain-resident cells. The decreasing level of CD47 releases macrophages from the inhibitory control, thereby increasing myelin phagocytosis [166].

Additionally, there is a study that compares RRMS patients with a group of progressive MS, including both SPMS and PPMS. Although the researchers did not split up those progressive phases, we would like to draw attention to their work because they detected miRNAs, whose combination improves discriminatory power between RRMS and progressive MS, and which might play a role in neuroinflammation and neurodegeneration in MS. Ebrahimkhani et al. profiled exosomal miRNAs and reported nine miRNAs (miR-15b-5p, miR-23a-3p, miR-223-3p, miR-374a-5p, miR-30b-5p, miR-433-3p, miR-485-3p, miR-342-3p, miR-432-5p), differently expressed in RRMS and progressive MS [167]. They showed that the combination of three miRNAs (miR-223-3p + miR-485-3p + miR-30b-5p) improves discriminatory power between RRMS and progressive MS with a 95% accuracy rate [167].

A recent study revealed that miR-485-3p might impact the differentiation and proliferation of NSCs to neuron and astrocytes cells, the activity of which is disrupted in neurodegenerative diseases. It was indicated that miR-485-3p targets thyroid receptor-interacting protein 6 (TRIP6) expression, which mediates signal transduction modulation during cell migration and adhesion, thereby diminishing proliferation and inducing differentiation of NSCs [168]. Yu et al. claimed that miR-485-3p might play a neurotoxic role while reducing neuronal viability and exacerbating neuroinflammation. Their experiments established that the knockdown of miR-485-3p promoted decreased cell proliferation and increased cell apoptosis induced by amyloid β-peptide in Alzheimer’s disease [169]. Studies performed on the BV2 microglial cells after lipopolysaccharide treatment exhibited that the reduction of miR-485-3p could inhibit inflammatory responses, which suggested the negative regulatory effects of miR-485-3p on neuroinflammation [170].

It was reported that downregulation of miR-30b-5p is necessary to generate fully functional macrophages and DCs. Its upregulation inhibits the release of inflammatory cytokines, such as TNF-α, IL-6, and IL-12, in lipopolysaccharide-stimulated cells in cultured and transfected cells [171]. The examination of the Kyoto Encyclopedia of Genes and Genomes (KEGG) database for miR-30b-5p, performed by Brennan et al., pointed out that this miRNA is able to target genes of pathways connected to neurodegeneration, including the Wnt pathway engaged in oligodendrocyte development and the remyelination process [172,173]. Zheng et al. demonstrated that miR-30b-5p is associated with a diminished expression level of the anti-apoptotic protein Bcl2 on the mouse pancreatic β-cell line [174]. It could be possible that this miRNA could also have an impact on Bcl2 expressed in peripheral lymphocytes whose dysregulated expression is a feature of clinically active MS [175]. Moreover, as proved by Zettl et al., chronic progressive MS patients exhibited a higher proportion of bcl-2-expressing T cells than patients with RRMS. In addition, active demyelinating lesions revealed a lower bcl-2-positive T cells number than remyelinating and demyelinated lesions. Therefore, this protein, expressed in MS plaques, might have important effects on the regulation of the persistence of the inflammatory cells in the CNS [176].

**Figure 2:**
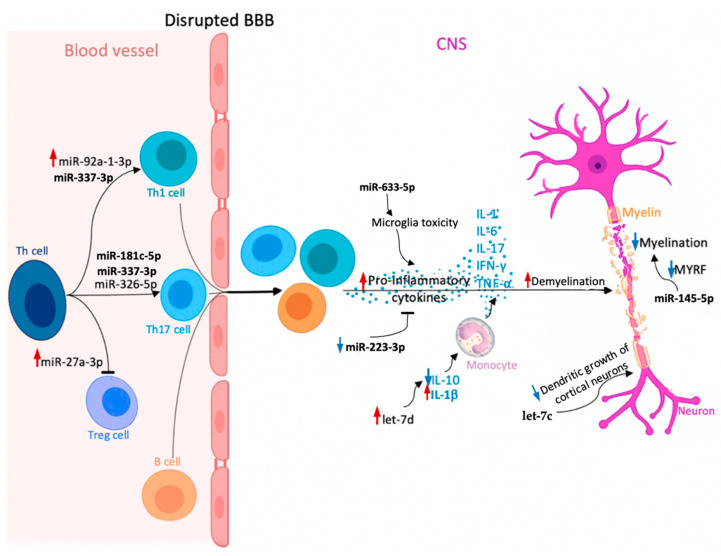
Potential role of circulating microRNAs (miRNAs) involved in multiple sclerosis (MS) pathogenesis, which could be useful in discriminating between relapsing-remitting multiple sclerosis (RRMS) and secondary progressive multiple sclerosis (SPMS) (miR-145-5p, miR-181c-5p, miR-223-3p, miR-27a-3p, miR-326-5p, miR-92a-1-3p, let-7c, let-7d, miR-337-3p, miR-633-5p). Upregulation and downregulation of specific miRNAs influence the Th cell differentiation into pro-inflammatory Th17 and Th1 cells, which together with B cells can cross the blood–brain barrier (BBB) and migrate to the central nervous system (CNS), where they proliferate and secrete pro-inflammatory cytokines (interleukin-1 (IL-1), IL-6, IL-17, interferon-γ (IFN-γ), and tumor necrosis factor α (TNF-α)). In the CNS, different miRNAs play various roles in cytokine secretion, monocyte stimulation, or neuronal maturation. As a consequence, dysregulation of these processes causes myelin sheath damage and, eventually, neuronal loss. Red arrow—upregulation of specific miRNA in MS compared to healthy controls; increased effect exerted by a given miRNA. Blue arrow—downregulation of specific miRNA in MS compared to healthy controls; decreased effect exerted by a given miRNA.

MiRNAs differentiating RR from SP described in this review are illustrated in Figure 2 and summarized in Table 1.

## 5. Limitations in miRNA Biomarker Studies

To summarize, available published studies point out that circulating miRNAs may have an important role as biomarkers in the diagnosis and prognosis of MS in future clinical practice. However, there are a small number of studies concerning circulating miRNAs’ expression in MS, especially in the context of miRNA profile difference between RRMS and SPMS subtypes. Furthermore, the research on circulating miRNAs as biomarkers is still in the early stage; thus, the findings usually lack reproducibility and are often even divergent. Studies evaluating circulating miRNAs have significant limitations, including the variability in biological sources and different techniques used to isolate and analyze (e.g., microarray, qRT-PCR, dPCR, and NGS) miRNAs. There are still no studies that analyze the differences in the level of miRNA expression measured in cell populations between RR and SP patients. Performing such an analysis would significantly narrow the pool of analyzed miRNA molecules to those which are cell-specific, and could constitute an important object in targeted therapies. Currently, there are studies that reported differences in miRNAs’ expression in different types of cells in each subtype of MS separately, in comparison to the control group. However, there are still not enough studies that analyze the level of miRNA expression in cells as a potential marker distinguishing the RR from the SP subtype. The next limitation is the lack of a large sample size and validation of results in independent cohorts, which is why the works with limited sample size and those with poor study design should be carefully considered. Another limitation factor of many original works is the fact that the authors do not specify the time of the biological fluids’ collection (at the diagnosis or at a different time point) and do not add the information on the treatment of patients. It would certainly allow us to take into consideration the potential influence of drugs on the level of miRNA and increase the reliability of the obtained results. Ultimately, one of the biggest hurdles facing researchers who want to determine the role of individual miRNAs in various disease entities is the complex network of relationships between the miRNAs and target transcripts. All those factors pose a huge problem as far as finding reliable and foolproof miRNAs, which might be characteristic for specific MS stage and effective prognosis of the RRMS-to-SPMS transition. Therefore, although some miRNAs might be useful during discrimination between RRMS and SPMS, there is a need to confirm their effectiveness via clinical trials.

## 6. Conclusions

Circulating miRNAs are particularly interesting molecules that may be able to identify differences in epigenetic regulation and pathophysiological mechanisms in different types of diseases. Research conducted on miRNAs in MS seems to be very promising. The findings suggest significant differences in the expression of many miRNAs between RRMS and SPMS subtypes. Currently, there is no sufficient data that would support the association of the expression level of specific circulating miRNA molecules with the development of pathological processes characteristic of particular stages of the MS course. While current immunomodulatory therapies have been shown to be efficacious in the early stages of MS, these therapies are less potent in the later phases of MS. Thus, distinguishing the conversion of RRMS to SPMS early is a great need. When the disease transits from the RR to the SP phase, the modification of the therapeutic protocol is required, ensuring a slower development of the disease by increasing the effectiveness of the treatment. Potential markers for RR-to-SP transition include: miR-92a-1-3p, let-7c, let-7d, miR-145-5p, miR-27a-3p, miR-337-3p, miR-633-5p, miR-181c-5p, and panels of miR-223-3p + miR-326-5p + miR-145-5p, miR-326-5p + miR-145-5p, and miR-223-3p + miR-326-5p. Nevertheless, the functions of most miRNAs in the pathophysiology of MS still remain largely unexplained. Currently, there are many miRNAs as potential markers of neuroinflammation and neurodegeneration, but further studies are desirable to verify those biomarkers as useful tools in clinical application, being novel therapeutic targets for treating MS. Each subtype of MS still requires its own specific miRNA profile.

## Figures and Tables

**Table 1:** Circulating miRNAs and combinations of miRNAs as potential tools in distinguishing RRMS from SPMS.

miRNA	Function	SPMS vs. RRMS	Biological Material	Patients Number	miRNAs Number Included in the Analysis	Ref.
miR-181c-5p	Regulates neuronal maturation and synaptogenesis in the cortex	Upregulated	CSF	81 RRMS 106 SPMS 0 HCs 211 OND	3	[118]
		Downregulated		17 RRMS 30 SPMS 0 HCs 39 OND	760	[117]
miR-633-5p	Targets mRNAs transcripts involved in neuroinflammatory pathways	Downregulated	CSF	17 RRMS 30 SPMS 0 HCs 39 OND	760	[117]
miR-27a-3p	Regulates the differentiation of Th1 and Th17 cells and the accumulation of Tregs Regulates oligodendrocytes differentiation	Downregulated	Serum	29 RRMS 19 SPMS 30 HCs	652	[92]
miR-337-3p	Initiates through Rap1 signaling pathogenic character of T cells	Downregulated	Serum	115 RRMS 51 SPMS 88 HCs	652	[141]
miR-92a-1-3p	Targets CD40-CD40L pathway Regulates cell cycle and cell signaling Promotes Th1 differentiation	Downregulated	Plasma	51 SPMS 50 RRMS 32 HCs	368	[104]
miR-145-5p	Regulates the expression of myelin gene regulatory factor					
let-7c	Plays a role in suppressing the dendritic growth of cortical neuron					
let-7d	Regulates immunological and inflammatory responses					
Combination of: miR-223-3p + miR-326-5p + miR-145-5p; miR-326-5p + miR-145-5p; miR-223-3p + miR-326-5p	miR-223-3p: Modulates the nuclear factor κB pathway Regulates inflammatory responses in macrophages	No statistical significance	Serum	18 RRMS 19 SPMS 23 HCs	3	[144]
	miR-326-5p: Regulates Th17 differentiation	Downregulated				
	miR-145-5p: Regulates the expression of myelin gene regulatory factor	No statistical significance				

All patients were untreated with disease-modifying therapies at the time of the sample collection, except the cohort of Kramer et al. (miR-181c-5p), which comprised both untreated patients and patients treated with azathioprine, interferons, glatiramer acetate, mitoxantrone, natalizumab, fingolimod, or fumaric acid. Detection technique: qRT-PCR. Abbreviations: other neurologic diseases—OND, relapsing–remitting multiple sclerosis—RRMS, secondary progressive multiple sclerosis—SPMS, healthy controls—HCs, cerebrospinal fluid—CSF.

## Data Availability

No new data were created or analyzed in this study. Data sharing is not applicable to this article.
